# Safety and efficacy of brachial approach for coronary angiography and percutaneous coronary intervention

**DOI:** 10.1186/s43044-024-00466-6

**Published:** 2024-03-26

**Authors:** Islam Ghanem, Mohamed Mesbah, Hesham Refaat

**Affiliations:** Cardiology Department, Zagazig University, Zagazig, Egypt

**Keywords:** Safety, Brachial access, Femoral access, Coronary intervention, Complications

## Abstract

**Background:**

There are many percutaneous coronary approaches. The most commonly used one is the radial artery because of its lowest risk of adverse vascular events. However, it could not be an option in some situations as congenital radial artery hypoplasia and spasm. In these cases, the second most common access is the femoral artery. The current literature over the brachial artery access is controversial. Thus, the aim of this study was to verify the brachial artery approach's effectiveness and safety.

**Results:**

We studied 300 patients who underwent elective coronary angiography and angioplasty in our institution with failed radial access between August 2022 and February 2023. They were classified into two groups; 150 patients with brachial access and 150 with femoral access. Access, procedural and fluoroscopy times were recorded. All patients were examined carefully immediately after the procedure and before discharge to assess any complications. Left brachial access was used more frequently than left femoral access (32.7% vs. 22.7%, *P* = 0.05), but no significant difference noted regarding right sided or bilateral access. Procedure time, fluoroscopy time, and contrast volume did not significantly differ (*P* = 0.19, 0.06 and 0.1 respectively). However, brachial group had shorter access time (2.6 ± 1.1 vs. 3.4 ± 0.7 min, *P* = 0.05) and hospital stay (3.5 ± 1.1 vs. 5.9 ± 1.3 days, *P*** < **0.001). Regarding major and minor complications (especially hematomas), they were significantly less in the brachial arm (*P* = 0.04 and *P* = 0.05, respectively).

**Conclusions:**

Brachial access is a safe, efficient and non-inferior to the femoral route for coronary intervention whenever radial access is not an option.

## Background

There are many feasible ways to reach the coronary arteries through a percutaneous technique as radial, femoral, ulnar, subclavian and also the extreme old approach through direct trans-lumbar aortic puncture [1]. And right now the most commonly used approach is the radial artery approach because it has the lowest risk of known vascular adverse events. This approach has many advantages as the patient can move freely directly after the procedure which helps in early discharge from the hospital with lower economic burden [2]. On the other hand, radial artery approach could not be an option as in the following situations: congenital radial artery hypoplasia, extreme tortuous course, abnormal radial artery origin, damaged arterial wall from previous catheterization and radial artery spasm. In these cases, the second most common access is through the femoral artery [3]. Previous studies have already discussed the brachial access regarding its technique, safety and resulting complications [4–6]. However, the current literature over the brachial artery access is controversial where some studies showed increased puncture site complications and others declared no higher complication rates than in patients with other arterial accesses [7]. The fact of this reported high complication rate of brachial artery approach is really not clear and necessitates more precise assessment to prove its safety and efficacy in relation to other arterial accesses, especially femoral one [8–10].

Thus, the efficacy of brachial access was the main issue of interest in our study by assessing procedural characteristics, access time, and hospital stay and comparing theses characteristics with those related to femoral access. Moreover, the complications resulting from brachial access were also our goal; either major or minor complications, to assess its safety compared to femoral access. So, we aimed in this study to shed light on brachial access and assess its safety and efficacy in comparison to femoral access done in patients admitted for coronary angiography and percutaneous coronary intervention (PCI) in our institution.

## Methods

### Study population

From August 2022 to February 2023, all patients who underwent elective scheduled coronary angiography and PCI in our institution with failed radial access were included in this retrospective cross-section study. The exclusion criteria were: ST segment elevation myocardial infarction (STEMI) patients underwent primary PCI (PPCI), patients who were in a cardiogenic shock requiring both inotropic support and intra-aortic balloon pump (IABP), and severely ill patients with a mechanical ventillatory support.

Finally, three hundred patients were finally enrolled and classified into two groups; 150 patients in a brachial access group and 150 patients in a femoral access group. In addition to demographic data, procedural characteristics, hospital stay, and complications were reported in both groups to assess safety and efficacy of the brachial access in relation to the femoral one. The study was carried out in adherence to the principles of the Declaration of Helsinki on Biomedical Research Involving Human Subjects. The institutional Ethics Committee approved the study protocol (ZU-IRB#10502). All study participants gave written informed consent before the procedure.

### Clinical data collection

Baseline demographic and clinical characteristics of all enrolled patients were obtained from hospital records and the cardiovascular risk factors were identified. The diagnosis of diabetes mellitus was confirmed in all patients receiving active treatment or defined as an abnormal glycated hemoglobin (> 6.5%), abnormal fasting blood glucose level (> 126 mg/dl) or abnormal 2 h postprandial level (> 200 mg/dl). Hypertensive patients were those receiving antihypertensive therapy or those with systolic blood pressure (SBP) > 140 mmHg and/or a diastolic blood pressure (DBP) > 90 mmHg. Dyslipidemia was defined if total cholesterol > 200 mg/dL or low-density lipoprotein cholesterol (LDL-C) > 100 mg/dL or when the patient was previously on lipid-lowering medication in accordance with Adult Treatment Panel III Guidelines [11]. Family history of CAD was defined as the presence of CAD in first-degree relatives before the age of 55 years for men and 65 years for women. Patients using tobacco products or those quit smoking within the past month were considered as smokers [12]. History of stroke, coronary artery bypass grafting (CABG), and peripheral vascular diseases were also included in clinical data collection.

### Blood samples and laboratory analysis

Venous blood samples were obtained from all patients prior to coronary angiography. The following parameters were obtained from all included patients: complete blood count (CBC), renal function tests (serum creatinine level and estimated glomerular filtration rate; eGFR), cardiac enzymes (Troponin I and creatine kinase-MB; CK-MB), and lipid profile.

### Electrocardiogram (ECG) analysis and Echocardiography protocol

A resting 12-lead ECG was obtained at a speed of 25 mm/s and a voltage of 10 mm/mV for all patients at admission. Transthoracic echocardiography (TTE) was done before coronary angiography by an experienced blinded cardiologist according to the current practice guidelines [13] using a commercially available device (Siemens ACUSON X300) with a 1.8 MHz phased array transducer to assess the diastolic function and LVEF by the modified Simpson’s rule.

### Coronary angiography and PCI protocol

Coronary angiography and PCI were performed by experienced operators according to standard protocols using Siemens (Axiom Sensis XP, Berlin, Germany) device at our catheterization laboratory via trans-brachial or trans-femoral approach using dedicated diagnostic and guiding catheters. Access, procedural and fluoroscopy times were recorded. All patients were examined carefully immediately after the procedure and before discharge to assess any complications. Complications were further classified into minor complications (conservative treatment only) and major complications (requiring surgical intervention).

### Brachial artery access (Fig. 1)

**Fig. 1:**
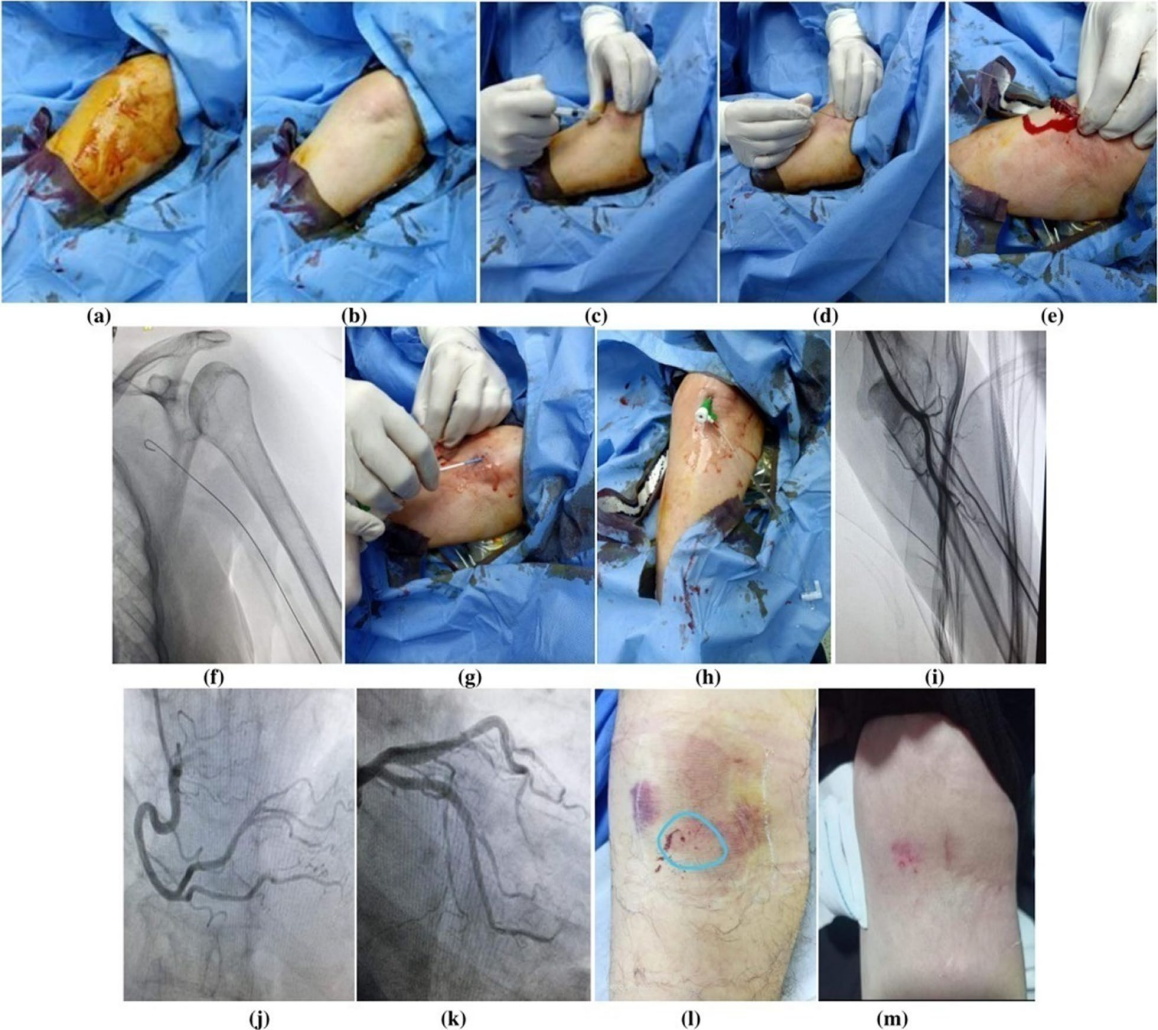
Steps of trans-brachial approach. a, b Proper sterile preparation of the antecubital fossa with a povidone iodine solution. c Subdermal injection of 2% xylocaine solution above the elbow crease. d The brachial artery punctureutilising either the single puncture approach or the modified Seldinger method. e, f Advancement of a 30 cm guidewire. g–i insertion of an arterial sheath over the wireand confirmed by fluoroscopy. j, k RCA and LMCA engagement via trans-brachial approach. l Removal of the arterial sheath followed by direct digital compression to the brachial artery for 10 min to achieve hemostasis. m Maintain the arm in such position for 4–6 h

Patients with unsuccessful radial access (both in the left and right radial arteries) and operators who lacked trans-ulnar approach experience or who lacked motivation to gain femoral access were the selection criteria for the brachial approach. In all cases, there was no failed femoral approach after failure to get the radial access as the femoral artery puncture had never been attempted. In case of congenital anomalies or extreme tortuosity of the arterial system resulting in failed radial approach, the brachial approach was abandoned in favour of the femoral one.

Brachial access is being performed more frequently with similar benefits compared to radial and ulnar arterial accesses; especially in case of not successfully getting radial artery access, but the vascular and neurological complications become hurdles to interventional cardiologists. Specifically, failed radial approach was related to unsuccessful radial access attempts due to its limitations including small diameter, spasm, tortuosity, anatomic variants, and asymptomatic occlusion. In addition, other limitations include an occluded radial artery restricting future cardiac catheterization, bypass grafts, and dialysis fistulae [14–16].

Depending on the pulse condition, the puncture side (left or right) was selected. It had never been necessary to use the Sones or brachial cut-down techniques. The antecubital fossa was draped in a sterile fashion after proper sterile preparation with a povidone iodine solution. After that, 2% xylocaine solution was injected sub-dermal above the elbow crease where the brachial pulsation was best palpated. The modified Seldinger technique or the single puncture technique was used to prick the brachial artery followed by advancing a 30 cm guidewire with a sheath over it. In case of coronary angioplasty, heparin (10,000 i.u) was given, otherwise only 5000 i.u was given in diagnostic procedures. Catheters and wires were used according to operator preference.

The arterial sheath was removed after the procedure and the brachial artery was then directly compressed for at least 10 min to establish hemostasis. After that, further local compression using an elastic bandage was applied to the brachial artery and the elbow immobilized by an arm board. The arm was kept in such position for 4 to 6 h.

### Femoral artery access

The selection criteria for femoral approach were patients with failed arm approach, and according to operators’ preference. Right femoral approach was used as a default approach. However, left side could be used in case of (a) weak right femoral pulse secondary to peripheral arterial disease, or (b) haemodialysis with insertion of double lumen catheter in the right femoral region. Both right and left sides were used in chronic total occlusion intervention.

The groin was draped after sterile preparation with a povidone-iodine solution. Under local anesthesia, percutaneous puncture of the femoral artery was done with an 18-G needle using either direct anterior puncture or modified Seldinger techniques. After that, a short 0.0359J-wire was passed with a sheath over it. For iliac negotiation, hydrophilic guidewires were employed and the operator's preferred catheter was used. Heparin dosage was administered in accordance with the type of procedure; 10,000 IU of heparin were administered during coronary angioplasty.

The arterial sheath was removed 4 to 6 h after the procedure. Manual digital pressure over the femoral artery was applied for 15 min to achieve hemostasis and then further local compression was applied by a sand bag and an elastic band for four hours. The patients were instructed to spend the entire process in bed and not to ambulate for 8 h. Doppler evaluation to both brachial and femoral arteries was done the day after the procedure.

### Efficacy and safety

In this study, the procedural characteristics were reported in both brachial and femoral accesses to assess the efficacy of brachial approach as an alternative arterial access. The procedural characteristics included procedure type including coronary angiography, simple PCI, chronic total occlusion PCI (CTO PCI), left main coronary artery PCI (LMCA PCI), bifurcation PCI, and intracoronary imaging in addition to sheat size (5, 6, and 7 french), amount of contrast, and access, procedural, fluoroscopic times. Also, hospital stay in days was also reported in both brachial and femoral accesses. All these data were documented in brachial access group to assess its efficacy in relation to femoral access group.

Regarding safety, access complications were categorized as major and minor. Major complications included moderate/severe bleeding at puncture or non-puncture sites associated with a significant hemoglobin drop and required blood transfusion, permanent neurological deficit in addition to vascular complications as thrombotic occlusion, abscess, fistula, and pseudo-aneurysm. Minor complications included minor bleeding not requiring blood transfusion, transient neurological deficit, and local hematoma not requiring surgery. All patients were re-evaluated both before discharge and during outpatient appointments.

### Statistical analyses

Data distribution was assessed according to the Kolgormonov-Smirnov test. Categorical data were compared using the chi-square test or Fisher exact test. Continuous variables were compared using an unpaired Student’s t-test or Mann–Whitney *U*-test. Data were expressed as mean ± standard deviation. All tests were two-sided, and a *p*-value of < 0.05 represented statistically significant differences. All analyses were performed using SPSS version 20 (SPSS Inc., USA).

## Results

From the database of our catheterization laboratory, we studied 300 patients who underwent coronary angiography and possible PCI between August 2022 and February 2023. Patients were categorized into two groups; 150 patients who underwent coronary angiography through brachial access and the other 150 patients who underwent coronary angiography through femoral access. There was no statistically significant difference between the studied groups regarding age, sex and body mass index.

Regarding cardiovascular risk factors, 142 (47.3%) of all patients were diabetic, 119 (49.7%) of all patients were hypertensive, 25 (8.3%) had positive family history of CAD, 180 (60%) were smokers and 38 (46%) were dyslipidemic. There was no statistically significant difference between both groups regarding cardiovascular risk factors, history of stroke, peripheral vascular diseasesnor previous CABG. Both groups had preserved systolic function. There was no statistically significant difference between both groups regarding other clinical and laboratory characteristics (Table 1).

**Table 1:** Baseline characteristics of the studied groups

	All patients (n = 300)	Brachial access (n = 150)	Femoral access (n = 150)	*P*-value
Demographic characteristics				
Male sex, n (%)	164 (54.7)	81 (54.0%)	83 (55.3%)	0.82
Age (years)	66.3 ± 8.9	67.9 ± 8.1	64.6 ± 9.9	0.51
BMI (kg/m^2^)	28.0 ± 4.6	28.2 ± 5.7	27.8 ± 4.6	0.93
Cardiovascular risk factors				
Diabetes mellitus	142 (47.3%)	70 (48.7%)	72 (48.0%)	0.82
Hypertension	149 (49.7%)	73 (48.7%)	76 (50.7%)	0.73
Family history of CADs	25 (8.3%)	14 (9.3%)	11 (7.3%)	0.53
Smoking	180 (60.0%)	92 (61.3%)	88 (58.7%)	0.64
7 Dyslipidemia	138 (46.0%)	68 (45.3%)	70 (46.7%)	0.82
History of stoke	33 (11.0%)	13 (8.7%)	20 (13.3%)	0.19
History of CABG	25 (8.3%)	9 (6.0%)	16 (10.7%)	0.14
History of confirmed PVD	56 (18.7%)	23 (15.3%)	33 (22.0%)	0.13
Clinical characteristics				
Blood pressure (mmHg)				
SBP	137.9 ± 28.1	139.1 ± 26.7	136.8 ± 35.4	0.94
DBP	81.3 ± 14.6	82.7 ± 16.5	79.9 ± 15.9	0.84
Heart rate (b/m)	86.2 ± 16.7	87.2 ± 18.8	85.2 ± 18.4	0.9
LVEF (%)	52.4 ± 5.8	53.0 ± 5.7	51.8 ± 6.1	0.65
LV diastolic dysfunction				
Grade 1	153 (51.0%)	74 (49.3%)	79 (52.7%)	0.77
Grade 2	112 (37.3%)	59 (39.3%)	53 (35.3%)	
Grade 3	35 (11.7%)	17 (11.3%)	18 (12%)	
Laboratory characteristics				
Hemoglobin (gm/dl)	12.7 ± 1.1	13.1 ± 0.9	12.5 ± 1.2	0.5
Leukocytes (× 10^3^/µL)	8.1 ± 1.5	7.6 ± 1.2	8.7 ± 1.6	0.23
Platelets (× 10^3^/µL)	226.5 ± 73.9	225.2 ± 73.4	231.1 ± 76.6	0.64
CK-MB (U/L)	205.4 ± 124.9	212.2 ± 142.4	198.6 ± 136.2	0.91
Troponin (ng/ml)	19.4 ± 13.1	20.8 ± 15.3	17.9 ± 13.8	0.82
TGs (mg/ml)	155.02 ± 55.59	156.14 ± 58.77	151.13 ± 43.19	0.58
LDL-C (mg/dl)	123.6 ± 21.1	124.7 ± 23.1	122.5 ± 23.9	0.91
HDL-C (mg/dl)	38.86 ± 7.61	38.91 ± 7.56	38.65 ± 7.87	0.83
Creatinine (mg/dl)	1.02 ± 0.47	1.03 ± 0.48	1.01 ± 0.47	0.82
eGFR, ml/min/1.73 m2 (%)	94.01 ± 42.37	91.37 ± 40.41	103.17 ± 47.89	0.13

*BMI* body mass index, *CABG* coronary artery bypass graft, *CADs* coronary artery diseases, *CK-MB* creatinine kinase-MB, *DBP* diastolic blood pressure, *eGFR* estimated glomerular filtration rate, *HDL-C* high density lipoprotein cholesterol, *LDL-C* low density lipoprotein cholesterol, *LV* left ventricle, *LVEF* left ventricular ejection fraction, *PVD* peripheral vascular diseases, *SBP* systolic blood pressure, *TGs* triglycerides

Both groups had similar rates of coronary angiography, simple PCI and complex procedures like CTO-PCI, LMCA-PCI and bifurcation PCI including intravascular imaging, thus both groups were well matched. Regarding technical details, there was no statistically significant difference between both groups regarding sheath size. Left brachial access was used more frequently than left femoral access in 49 (32.7%) versus 34 (22.7%) patients (*P* = 0.05), but no significant difference regarding right sided or bilateral access. This is explained by right arm deformity in 15 patients, 10 patients had recently fixed right arm fractures, 3 patients had burns in right arm, 2 patients had inserted Swan Janz catheters via right antecubital vein, 7 patients had inserted right antecubital vein cannula limiting the space for inserting arterial sheath, left brachial spasm in 2 female patients as we don’t use spasmolytics routinely for brachial approach but we keep it only bailout and 10 patients on their request for fear of developing right arm neurological or vascular deficits because they perform fine-tuning hand-skilled occupations.

**Fig. 2:**
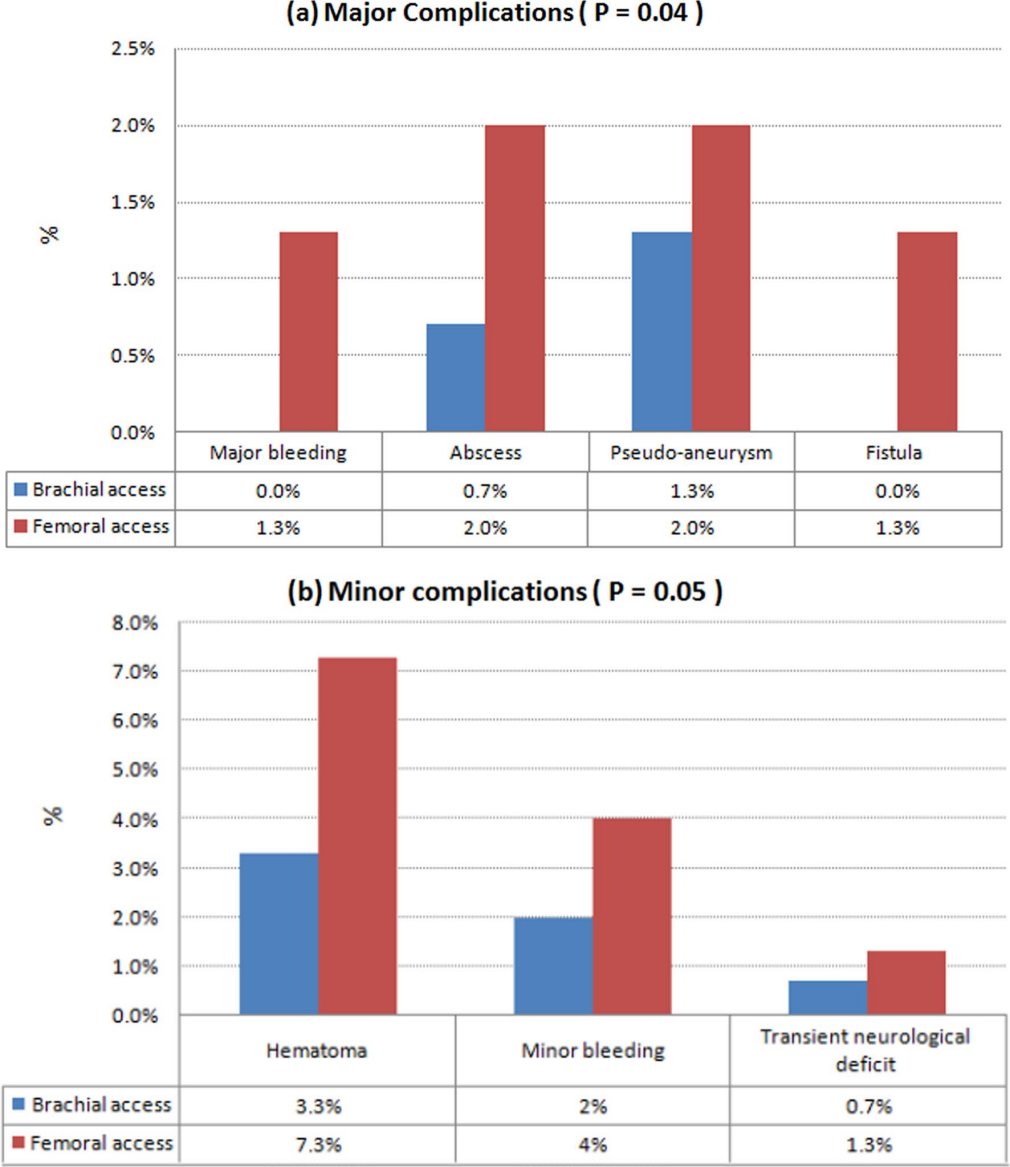
Complications resulting from brachial and femoral accesses. **a** Major complications including major bleeding, abscess, pseudo-aneurysm and fistula were statistically significant in the femoral access compared to the brachial access (*P* = 0.04). **d** Minor complications including minor bleeding, hematoma and transient neurological deficit were also statistically significant in the femoral access compared to the brachial access (*P* = 0.05)

Procedure time, fluoroscopy time and contrast volume did not change in a way that was statistically significant (*P* = 0.19, 0.06 and 0.1 respectively). Brachial group had shorter access time (2.6 ± 1.1 vs. 3.4 ± 0.7 min, *P* = 0.05) and shorter hospital stay (3.5 ± 1.1 vs. 5.9 ± 1.3 days, *P*** < **0.001) (Table 2). Regarding major complications, they were significantly less in the brachial arm (*P* = 0.04), 2 patients in femoral group had bleeding required blood transfusion. Regarding minor complications, they were significantly less in the brachial arm (*P* = 0.05), especially hematomas were less in brachial group in 5 patients (3.3%) versus 11 patients in femoral group (7.3%) (Table 3) (Fig. 2).

**Table 2:** Procedural characteristics of the studied groups

	All patients (n = 300)	Brachial access (n = 150)	Femoral access (n = 150)	*P*-value
Procedural characteristics				
Procedure				0.96
Coronary angiography (%)	141 (47.0%)	73 (48.7%)	68 (45.3%)	
Simple PCI (%)	83 (27.7%)	40 (26.7%)	43 (28.7%)	
CTO PCI (%)	10 (3.3%)	4 (2.7%)	6 (4.0%)	
LMCA PCI (%)	24 (8.0%)	11 (7.3%)	13 (8.7%)	
Bifurcation PCI (%)	33 (11.0%)	17 (11.3%)	16 (10.7%)	
Intracoronary imaging (%)	9 (3.0%)	5 (3.3%)	4 (2.7%)	
Sheath size				
5F (%)	20 (6.7%)	11 (7.3%)	9 (6.0%)	0.6
6F (%)	267 (89.0%)	133 (88.7%)	134 (89.3%)	0.85
7F (%)	13 (4.3%)	6 (4.0%)	7 (4.7%)	0.78
Side				
Right (%)	207 (69.0%)	97 (64.7%)	110 (73.3%)	0.11
Left (%)	83 (27.7%)	49 (32.7%)	34 (22.7%)	0.05*
Both left and right (%)	10 (3.3%)	4 (2.7%)	6 (4.0%)	0.52
Access time (min)	3.0 ± 0.9	2.6 ± 1.1	3.4 ± 0.7	0.05*
Procedure time (min)	66.2 ± 24.1	60.8 ± 26.2	72.4 ± 20.6	0.19
Fluoroscopy time (min)	26.2 ± 4.8	23.9 ± 5.0	29.6 ± 1.1	0.06
Amount of contrast (ml)	169.5 ± 68.1	133.9 ± 55.6	205.1 ± 64.7	0.1
Hospital stay (days)	4.6 ± 1.7	3.5 ± 1.1	5.9 ± 1.3	< 0.001*

*CTO* chronic total occlusion, *F* french size, *LMCA* left main coronary artery, *PCI* percutaneous coronary intervention

*statistically significant difference

**Table 3:** The Incidence of complications in the studied groups

	All patients (n = 300)	Brachial access (n = 150)	Femoral access (n = 150)	*P*-value
Major complications	13 (4.3%)	3 (2.0%)	10 (6.7%)	0.04*
Arterial thrombosis/occlusion (%)	–	–	–	–
Major bleeding required blood transfusion (%)	2 (0.7%)	0 (0%)	2 (1.3%)	0.16
Abscess (%)	4 (1.3%)	1 (0.7%)	3 (2.0%)	0.31
Pseudo-aneurysm (%)	5 (1.7%)	2 (1.3%)	3 (2.0%)	0.65
Fistula (%)	2 (0.7%)	0 (0%)	2 (1.3%)	0.16
Permenant neurological deficit (%)	–	–	–	–
Minor complications	28 (9.3%)	9 (6.0%)	19 (12.7%)	0.05*
Hematoma (%)	16 (5.3%)	5 (3.3%)	11 (7.3%)	0.16
Minor bleeding (%)	9 (3.0%)	3 (2.0%)	6 (4.0%)	0.31
Transient neurological deficit (%)	3 (1.0%)	1 (0.7%)	2 (1.3%)	0.56

*statistically significant difference

## Discussion

The main results of our research was that we can use brachial approach as a second line after failing radial access instead of femoral access with shorter access time, capability to use large sheath size and perform complex procedures with equal or even low complication rate compared to femoral approach.

Since Campeau's 1989 description of the first transradial access, a steadily rising interest in this technique developed, particularly in cardiac and more lately, in neuro and body-interventions [17]. After randomized trials revealed significantly lower puncture site bleeding complications when using sheath sizes of up to 7F, as well as reduced all-cause mortality [18, 19], the American Heart Association (AHA) updated their recommendation to a "radial first" strategy due to level I evidence [20, 21]. However, in some circumstances, this is challenging because of the small arterial size, the necessity to install a bigger sheath, radial artery spasm, radial artery blockage due to repeated interventions through the same access, as well as radial artery tortuosity or anomalies [22–24]. In these scenarios, we shift to another access as the contralateral radial or femoral access.

Safety and efficacy of brachial approach for percutaneous peripheral intervention was confirmed by Basche et al. [25] and Aschenbach et al. [26]. Accordingly, we performed this study to assess if we can depend on the brachial access as an alternative to femoral access in those situations or not. We had 150 patients done through brachial approach and 150 patients done through femoral approach, both were second line after failed radial access. Brachial puncturing was successful in all studied cases without need for turn-over to another access, this was in agreement with Basche et al. [25] and Gan et al. [8] who had successful brachial puncturing in more than 99% of patients studied, this means it isn’t a difficult approach to use. Both groups in our study had similar demographic characteristics and preserved systolic function, thus both groups were well matched.

We were able to perform complex procedures like CTO, left main coronary and bifurcation lesions intervention through the brachial access. Also, we used intravascular coronary imaging in 5 patients (3.3%) through brachial approach. The 6F sheath was the most frequently used sheath size in both groups, but we could use also 7F sheath in the brachial group in six patients (4.0%). Right side was the default for brachial and femoral approaches, but significantly left brachial was used more than left femoral access in 49 versus 34 patients (32.7% vs. 22.7%, *P* = 0.05), as previously mentioned.

No prolongation of fluoroscopy or procedure time was observed in the brachial group, also no need for more contrast volume (*P* = 0.1), on the other hand, brachial group had shorter access time (2.6 ± 1.1 vs. 3.4 ± 0.7 min, *P* = 0.05) due to extensive experience of our centre regarding trans-arm procedures (radial and ulnar approaches). One of the major findings was shorter hospital stay in the brachial versus femoral group (3.5 ± 1.1 days vs. 5.9 ± 1.3 days, *P* < 0.001) as we removed the brachial sheaths by manual compression bandages one hour post-PCI versus six hours post-PCI in the femoral group. Immediate mobilization and early (4 h) hospital discharge of elective smooth cases post-PCI compared to bed stay and later (6–8 h) hospital discharge in the femoral group allowed more patient satisfaction especially in females who mostly prefer trans-arm rather than trans-femoral procedures and also less burden on recovery beds.

Regarding safety of the brachial access, major complications were significantly less in the brachial arm (*P* = 0.04), 2 patients in femoral group had bleeding required blood transfusion, both had 7F sheath and had uninterrupted oral anticoagulation before the procedures in agreement with Hildick-Smith et al. [27], who found 20% risk for major vascular hazards in patients with peripheral vascular disease when using larger sheaths. Also, Kret et al. [5] found positive correlation between sheath size and access complications. No single patient developed arterial thrombosis or occlusion in both groups; in agreement with Gan et al. [8].

Regarding minor complications, they were significantly less in the brachial arm (*P* = 0.05), especially hematomas were less in brachial group, 5 (3.3%) vs. 11 (7.3%) patients. Transient neurological deficit like numbness occurred in one patient in brachial and two patients in femoral group, but no permanent neurological deficit occurred in both groups.

Complication rate of brachial access was reported high up to 36% in some studies discouraging the operators to rely on that approach [8, 28]. But some studies reported the rate up to 1.3% to 3.4% [29]. We open the door again encouraging the use of brachial approach with a low complication rate both major (2%) and minor (6%) complications.

In agreement with Alvarez-Tostado et al. [28], compared to femoral technique, brachial approach could be utilized with an equal or even lower complication rate where we had lower major (2% vs. 6.7%) and minor complications (6% vs. 12.7%). This could be attributed to our expertise in arm approach, proper caring of patients with high complication risks as anticoagulated patients, proper hemostasis (10 min manual compression after sheath removal) and proper follow up of the haemostatic bandage by nursing team.

In agreement with Armstrong et al. [29] and Franz et al. [30], early mobilization, patient comfort especially patients who cann`t tolerate lying on back for long duration as patients with vertebral disc problems and with heart failure, suitability for patients with occlusive aortoiliac disease and saving some catheter length in taller patients favor the brachial approach. On the other hand, need for routine heparinization due to small brachial artery caliber, difficulty of catheter engagement in some instances, poor catheter support are the procedure ʼ s cons.

### Limitations

The main limitation was that brachial procedures were done by experienced radialistis excluding operators in different learning curves. If we need to get a global overview of the safety and efficacy of the procedure, we had to include operators from different centers with different levels of experience regarding arm approaches. Also, few number of complex-highly indicated patients (CHIP) needing rotablation with different size burrs and frequent runs of intravascular imaging. Other limitations were small sample size and that it was a single centre study. Thus, larger multicenter blinded studies will be required in the future to avoid operator`s bias and to better assess the clinical impact, safety, and efficacy of the brachial approach. Finally, complications should be monitored and assessed with a Doppler study to evaluate long term adverse vascular outcomes.

## Conclusions

Brachial approach, performed by operators experienced in radial access, is a secure and effective substitute for the femoral approach for coronary intervention when radial access is not a possibility as in patients with small, anomalous radial artery, patients with arterio-venous shunts on dialysis or to preserve the radial artery as an arterial graft in patients scheduled for CABG. Larger multicentre studies will be required to prove the safety and efficacy of this brachial approach as an alternative to the femoral approach in such patients.

## Data Availability

The datasets used and/or analysed during the current study are available from the corresponding author on reasonable request.

## Abbreviations

AHA: American heart association
BMI: Body mass index
CABG: Coronary artery bypass grafting
CAD: Coronary artery disease
CBC: Complete blood count
CHIP: Complex-highly indicated patients
CK-MB: Creatine kinase-MB
CTO: Chronic total occlusion
DBP: Diastolic blood pressure
ECG: Electrocardiogram
eGFR: Estimated glomerular filtration rate
F: French size
G: Gauge
HDL-C: High density lipoprotein cholesterol
IABP: Intra-aortic balloon pump
LDL-C: Low density lipoprotein cholesterol
LMCA: Left main coronary artery
LVEF: Left ventricular ejection fraction
PCI: Percutaneous coronary intervention
PPCI: Primary percutaneous coronary intervention
PVD: Peripheral vascular diseases
SBP: Systolic blood pressure
STEMI: ST segment elevation myocardial infarction
TGs: Triglycerides.
TTE: Trans-thoracic echocardiogram
